# Postoperative radiotherapy for completely resected Masaoka stage III thymoma: a retrospective study of 65 cases from a single institution

**DOI:** 10.1186/1748-717X-8-199

**Published:** 2013-08-13

**Authors:** Chengcheng Fan, Qinfu Feng, Yidong Chen, Yirui Zhai, Zongmei Zhou, Dongfu Chen, Zefen Xiao, Hongxing Zhang, Jian Li, Zhouguang Hui, Jun Liang, Jima Lv, Yousheng Mao, Luhua Wang, Jie He

**Affiliations:** 1 Department of Radiation Oncology, Cancer Hospital and Institute, Chinese Academy of Medical Sciences and Peking Union Medical College, Pan jia yuan nan li 17#, Chao Yang District, Beijing 10021, China; 2 Department of Thoracic Surgery, Cancer Hospital and Institute, Chinese Academy of Medical Sciences and Peking Union Medical College, Pan jia yuan nan li 17#, Chao Yang District, Beijing 10021, China; 3 Department of Radiation Oncology, Beijing Shijitan Hospital, 10#, Yangfangdian Tieyiyuan Road, HaiDian District, Beijing 100038, China

**Keywords:** Thymoma, Radiation, Surgery, Failure pattern

## Abstract

**Background:**

The role of adjuvant radiotherapy (RT) for patients with stage III thymoma after complete resection is not definite. Some authors have advocated postoperative RT after complete tumor resection, but some others suggested observation. In this study, we retrospectively evaluated the effect of postoperative RT on survival as well as tumor control in patients with Masaoka stage III thymoma.

**Methods:**

Between June 1982 and December 2010, 65 patients who underwent complete resection of stage III thymoma entered the study. Fifty-three patients had adjuvant RT after surgery (S + R) and 12 had surgery only (S alone). Of patients who had adjuvant RT, 28 had three-dimensional conformal RT (3D-CRT)/intensity modulated RT (IMRT) and 25 had conventional RT. A median prescribed dose of 56 Gy (range, 28–60 Gy) was given.

**Results:**

The median follow-up time was 50 months (range, 5–360 months). Five- and 10-year overall survival (OS) rates were 91.7% and 71.6%, respectively, for S + R and 81.5% and 65.2% for S alone (P = 0.5), respectively. In the subgroup analysis, patients with 3D-CRT/IMRT showed a trend of improved 5-year OS rate compared with conventional RT (100% vs. 86.9%, P =0.12). Compared with S alone, the 5-year OS rate was significantly improved (100% vs. 81.5%, P = 0.049). Relapses occurred in 15 patients (23.1%). There was a trend of lower crude local recurrence rates for S + R (3.8%) compared with S alone (16.7%) (P = 0.09), whereas the crude regional recurrence rates were similar (P = 0.9). No clear dose–response relationship was found according to prescribed doses.

**Conclusions:**

Adjuvant 3D-CRT/IMRT showed potential advantages in improving survival and reducing relapse in patients with stage III thymoma after complete resection, whereas adjuvant RT did not significantly improve survival or reduce recurrence for the cohort as a whole. Doses of ≤ 50 Gy may be effective and could be prescribed for adjuvant RT. To confirm the role of adjuvant 3D-CRT/IMRT in patients who undergo a complete resection of thymoma, a multicenter randomized study should be performed.

## Background

Although thymoma is rare, it is one of the most common mediastinal neoplasms and surgery remains the main therapeutic modality. Radiotherapy (RT) has also been recommended as an adjuvant therapy to reduce the rate of local and regional failure after incomplete or complete resection [1-4]. This is especially true in Masaoka stage III thymomas because of the involvement of surrounding vital structures [2,3,5]. However, the optimal management of these tumors remains unknown. Three-dimensional conformal RT (3D-CRT)/intensity modulated RT (IMRT) has been used to treat tumors with reduced damage to normal tissues and increased tumor control and survival rates. Therefore, further investigation into the value of adjuvant RT is warranted, especially with the use of 3D-CRT/IMRT for stage III thymoma after complete resection. Here, we reviewed our data for patients with Masaoka stage III thymomas after complete tumor resection to determine the role of adjuvant RT.

## Methods

### Patient population

Between June 1982 and December 2010, 119 patients with stage III thymoma according to the Masaoka and modified Masaoka staging system [6,7] were treated at the Cancer Hospital of the Peking Union Medical College, Chinese Academy of Medical Science.

Of these patients, 70 underwent complete tumor resection; one patients were excluded from the study due to incomplete records and the other 4 were excluded due to preoperative RT followed by surgery and postoperative RT. The remaining 65 patients were enrolled for the analysis. This retrospective study was approved by our Institutional Review Board. The dates of follow-up appointments were obtained through a review of the hospital records, in addition to telephone, interviews and patient consultations. Patients with thymic carcinoma or carcinoid were excluded from this study.

### Pathologic review

Due to the possibility of invasion into surrounding structures, such as blood vessels, pericardium and lung, surgical notes were reviewed carefully to determine the completeness of tumor resection. Adhesion of the tumor to adjacent structures was not considered as confirmed invasion. Pathology reports were obtained and reviewed for all patients. Slides of surgical specimens were available for 63 of the 65 patients; these were reviewed by an experienced pathologist and classified as type A (*n* = 2), type B1 (*n* = 12), type B2 (*n* = 28), type B3 (*n* = 14) or type AB (*n* = 7) according to the WHO histopathological classification [8].

### Surgery

All of the 65 patients underwent complete tumor resection with negative surgical margins, including pericardiectomy, wedge resection of the lung, phrenicectomy or vein resection followed by replacement surgery when these surrounding structures were involved. Twelve patients (18.5%) received surgery only (S alone), and 53 patients (81.5%) received adjuvant RT after surgery (S + R).

### Radiotherapy

Of the patients who received postoperative RT, 25 patients had conventional RT and 28 patients had 3D-CRT/IMRT (6 patients with 3D-CRT, 22 patients with IMRT). The selection of patients for adjuvant RT was based principally on the surgeons’ judgments.

The radiation target volume was defined using preoperative images to identify the tumor extent or using metal marks placed during surgery. Fifty-one patients were treated with involved field, whose clinical target volume (CTV) was defined as tumor bed with margins extension of 1–2 cm; only 2 patients were treated with extended field, whose CTV was defined as the whole mediastinum with the upper and lower margins at the thoracic inlet and the diaphragmatic crurae, respectively. Both planning target volume (PTV) of involved field and extended field were defined as CTV plus a 0.5-1 cm margin.

A median prescribed dose of 56 Gy to PTV (range, 28–60 Gy) was given in 2-Gy daily fractions. Three patients received less than 30 Gy because of severe toxicities (2 patients) or unknown reason (one patient). Conventional RT was delivered with 6 or 8-MV photons from linear accelerators using an anteroposterior opposed field or angled anterior fields, with the spinal cord dose limited to 45 Gy. Two anterior, wedged portals or off-cord, oblique, opposed portals were often used to provide a boost to the anterior mediastinum to higher doses. Patients who were administered with 3D-CRT or IMRT all underwent computed tomography (CT) simulation (slice thickness of 5 mm) while immobilized in the supine position using a thermoplastic resin shells. Doses were delivered using a step-and-shoot technique with 6-MV photons from a linear accelerator with the forward (3D-CRT) or the inverse (IMRT) Pinnacle^3^ planning systems (Philips Healthcare, Madison, WI, USA), and at least 95% PTV received the prescribed dose.

### Chemotherapy

Two patients received adjuvant chemotherapy after surgery and RT. One was given two cycles of gemcitabine and cisplatin after resection; the regimen of the other patient was unknown.

### Follow-up

The last follow-up date was June 25th, 2012. Physical examination, abdominal ultrasonography, chest radiography and/or chest CT was performed every 3 or 6 months after treatment. The median follow-up time was 50 months (range, 5–360 months). Survival was calculated from the date of surgery. Disease-free survival (DFS) refers to the time from surgery to recurrence, disease-specific survival (DSS) refers to the time from surgery to tumor-induced death, and overall survival (OS) refers to the time from surgery to death from any cause.

### Statistical analysis

All analyses were conducted using SPSS 17.0 statistical software (SPSS, Chicago, IL, USA). The Kaplan–Meier method was used to estimate the survival rates and relapse rates, and the log rank test was used to test the difference between groups. The distribution of categorical variables in the two groups was tested using Fisher’s exact test. A P value of less than 0.05 was considered statistically significant.

## Results

### Patient characteristics

Twenty-two patients had accompanying symptoms of myasthenia gravis (MG) and two had pure red cell aplasia. The invasion of surrounding structures was as follows: lung in 29 patients, pericardium in 44, large veins in 18, large arteries in 3 and phrenic nerve in 18. In some patients, multiple structures were involved. Patient characteristics were similar between those who had adjuvant RT and those who underwent surgery alone (Table 1).

**Table 1:** Characteristics of patients

**Characteristic**	**S + R**	**S alone**	**χ**^**2**^	**P**
	**n = 53(%)**	**n = 12(%)**		
Sex			0.3	0.6
Male	26(49.1%)	7 (58.3%)		
Female	27(50.9%)	5 (41.7%)		
Age			0.0	0.9
>48	22(41.5%)	5 (41.7%)		
≤ 48	31(58.5%)	7 (58.3%)		
pathologic type			0.4	0.5
B3	13(24.5%)	4 (33.3%)		
Other subtypes	40(75.5%)	8 (66.7%)		
Tumor size			0.4	0.8
≤ 7cm	25(49.1%)	5 (41.7%)		
>7cm	28(50.9%)	7 (58.3%)		
Myasthenia Gravis			0.007	0.9
Presence	17(32.1%)	4 (33.3%)		
Absence	36(67.9%)	8 (66.7%)		
Multistructure invasion			0.003	0.9
Presence	26(49.1%)	6 (50.0%)		
Absence	27(50.9%)	6 (50.0%)		
Pleural effusion			1.5	0.2
Presence	6(11.3%)	0 (0.0%)		
Absence	47(88.7%)	12 (100.0%)		

S + R indicates surgery and radiotherapy; *S*, surgery.

### Survival

By the last follow-up date, 12 (18.5%) patients had died. Six died of tumor relapse, one from second primary malignant tumor, one from lung fibrosis caused by severe radiation pneumonitis, one from MG and three for unrelated reasons. The 5- and 10-year OS rates were 89.7% (95% confidential interval [CI], 85.1-94.3%) and 70.3% (95% CI, 61–79.6%), respectively, the 5- and 10-yr DFS were 76.9% (95% CI, 70.1-83.7%) and 53.2% (95% CI, 43.1-63.3%), respectively, and the 5- and 10-year DSS were 96.0% (95% CI, 93.2-98.8%) and 80.3% (95% CI, 71.6-89%), respectively.

**Figure 1:**
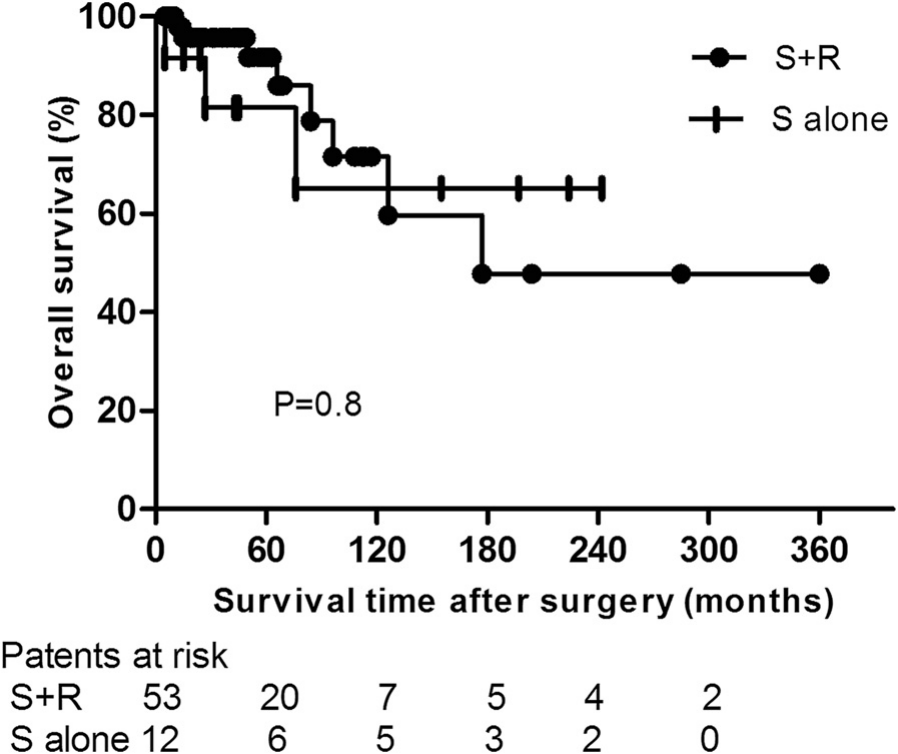
Comparison of overall survival between surgery and adjuvant radiotherapy (S + R) and surgery alone (S alone) groups.

**Figure 2:**
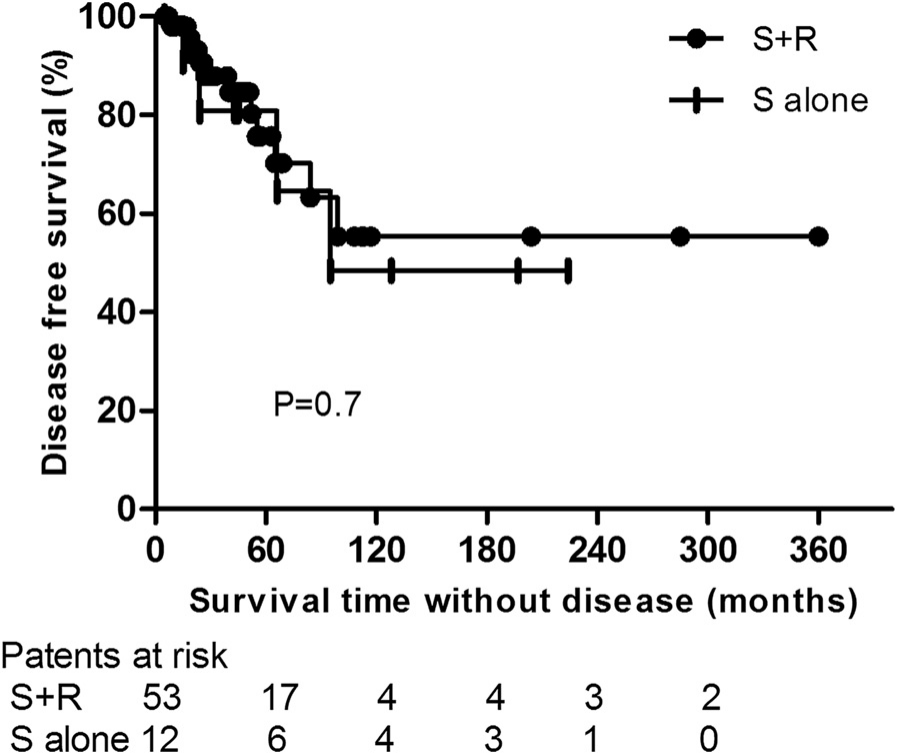
Comparison of disease-free survival between surgery and adjuvant radiotherapy (S + R) and surgery alone (S alone) groups.

**Figure 3:**
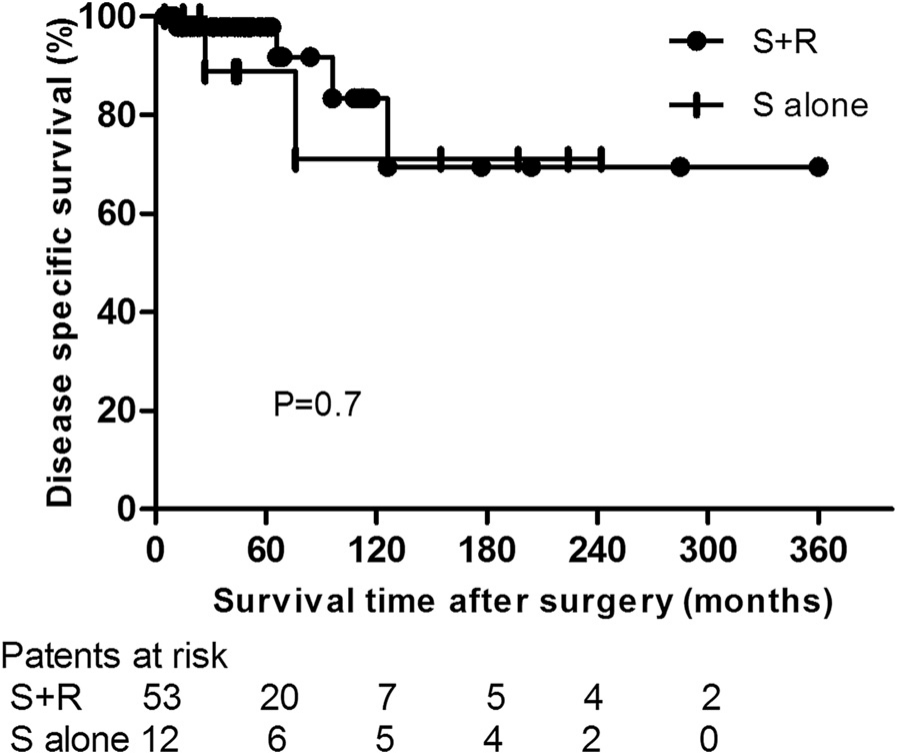
Comparison of disease-specific survival between surgery and adjuvant radiotherapy (S + R) and surgery alone (S alone) groups.

Five- and 10-yr OS rates were 91.7% (95% CI, 86.9-96.5%) and 71.6% (95% CI, 60.6-82.6%) in the S + R group and 81.5% (95% CI, 70.6-93.4%) and 65.2% (95% CI, 47.8-82.6%) in the S alone group (P =0.8; Hazard ratio [HR], 0.89; 95% CI, 0.22-3.5) (Figure 1). Five- and 10-yr DFS rates were 75.7% (95% CI, 67.8-83.6%) and 55.4% (95% CI, 43.6-67.2%) in the S + R group and 80.8% (95% CI, 68.6-93%) and 48.5% (95% CI, 29.3-67.7%) in the S alone group (P =0.7; HR, 0.78; 95% CI, 0.23-2.6) (Figure 2). Five- and 10-yr DSS rates were 97.9% (95% CI, 95.8-100%) and 83.4% (95% CI, 73.6-93.2%) in the S + R group and 88.9% (95% CI, 78.4-99.4%) and 71.1% (95% CI, 53.1-89.1%) in the S alone group (P =0.7; HR, 0.7; 95% CI, 0.11-4.4) (Figure 3).

In subgroup analysis, patients with 3D-CRT/IMRT had a trend of improved 5-yr OS rate (100%) compared with conventional RT (86.9% [95% CI, 79.8-94%]) (P = 0.12; HR, 4.7; 95% CI, 0.66-33.61), and a improved 5-yr OS rate when in pairwise compared with S alone (P = 0.049). Difference of OS rates was not statistically significant when these three subgroups were compared in pooled (P = 0.28). The 5-yr DFS and 5-yr DSS rates for patients with 3D-CRT/IMRT and conventional RT were 73.8% (95% CI, 64.5-83.1%) and 67.5% (95% CI, 46.8-88.2%) (P = 0.35; HR, 1.9; 95% CI, 0.49-7.38), and 100% and 96% (95% CI, 92.1-99.9%) (P = 0.28; HR, 4.7; 95% CI, 0.28-7.8), respectively. When compared in pooled with S alone, the difference of 5-yr DFS and DSS rates were not statistically significant either (P = 0.6 and P = 0.5, respectively). No patients treated with 3D-CRT or IMRT died till the date of follow-up, and patients with IMRT showed a similar 5-yr DFS rate compared with patients with 3D-CRT (80% [95% CI, 62.1-97.9%] vs.75% [95% CI, 53.3-96.7%], P = 0.32).

### Recurrence and salvage treatment

During the follow-up period, 15 patients (23.1%) had relapses (Table 2). Four had local recurrence (tumor bed or in-field recurrence) at the first recurrence, ten had regional recurrence (out-field recurrence) at the first recurrence (including chest wall, pleural, mediastinum, diaphragm and lung) and one had both regional recurrence (chest wall) and distant metastasis (bone) at first recurrence simutaneously. The median time to recurrence was 40 months (range, 9–99 months).

**Table 2:** Recurrence rates and sites between patients with surgery followed by adjuvant radiotherapy and surgery alone

**Therapy**	**No.of patients**	**No. of LR (%)**	**No. of RR (%)**	**First recurrence sites (%)**					
				**PL**	**MS**	**CW**	**Lung**	**DP**	**DM**
S + R	53	2 (3.8)	9 (17.0)	5 (9.4)	2 (3.8)	2 (3.8)	1 (1.9)	0	1 (1.9)
S alone	12	2 (16.7)	2 (16.7)	1 (8.3)	0	0	1 (8.3)	1 (8.3)	0

S + R indicates surgery plus radiotherapy, *S*, surgery; *LR*, local recurrence; *RR*, regional recurrence; *PL*, pleura; *MS*, mediastinum; *CW*, chest wall; *DP*, diaphragm; *DM*, distant metastasis.

From Table 2, it can be seen that there was a trend of lower crude local recurrence rates for S + R (3.8%) compared with S alone (16.7%) (P = 0.09), whereas the crude regional recurrence were similar (P = 0.9). The first site of recurrence for different radiation techniques was listed in Table 3. Conventional RT seemed to have more out-field recurrence compared with 3D-CRT/IMRT, which yet needs further follow-up for the shorter median follow-up time in patients with 3D-CRT/ IMRT.

**Table 3:** Recurrence rates and sites in patients with different radiotherapy mode

**RT mode**	**No.of patients**	**No. of IFR (%)**	**No. of OFR (%)**	**First recurrence sites (%)**				
				**PL**	**MS**	**CW**	**Lung**	**DP**
**Dose**								
≤ 50 Gy	20	1 (5)	5 (25)	3 (15)	1 (5)	1 (5)	1 (5)	1 (5)
> 50Gy	31	1 (3.2)	4 (12.9)	2 (6.4)	1 (3.2)	1 (3.2)	0	0
**Technique**								0
Conventional RT	25	1 (4)	8 (32)	4 (16)	2 (8)	2 (8)	1 (3.6)	1 (3.6)
3D-CRT/IMRT	28	1 (3.6)	1 (3.6)	1 (3.6)	0	0	0	0

RT indicates radiotherapy; 3D-CRT/IMRT, three dimensional conformal radiotherapy or intensity modulated radiotherapy; *IFR*, in-field recurrence; *OFR*, out-field recurrence; *PL*, pleura; *MS*, mediastinum; *CW*, chest wall; *DP*, diaphragm.

Eight patients underwent salvage treatment and seven received no further treatment after tumor recurrence. Five patients survived disease-free after salvage treatment; two of these underwent surgical resection, two had RT, and one received sequential chemotherapy and RT. The survival time after recurrence was 13–300 months. Two patients survived with disease after RT alone and one with sequential chemotherapy and RT. Two died of tumor relapse at 24 and 60 months after salvage retreatment and the other has been living with tumor for 27 months. Of the patients without salvage treatment, three patients died of tumor at 3, 5 and 12 months and the other four have been living with their tumor (median survival, 13 months) since the relapse was diagnosed.

### Radiation dose–response relationship

Fifty-one patients for whom radiation doses were known were subjected to further analysis. The number of patients given doses of ≤ 50 Gy and >50 Gy were 20 and 31, respectively. The 5-yr and 10-yr OS rates for patients given doses of ≤ 50 Gy were 94.7% (95% CI, 89.6-89.8%) and 65% (95% CI, 47-83%), and 88.8% (95% CI, 80.9-96.7%) and 58.2% (95% CI, 39–77.4%) for patients given >50 Gy (P = 0.7; HR, 0.7; 95% CI, 0.16-3.15). The 5-yr and 10-yr DFS rates were 73.9% (95% CI, 62.1-85.7%) and 46.2% (95% CI, 28.8-63.6%) for patients given doses of ≤ 50 Gy, and 73.2% (95% CI, 60.7-85.7%) and 54.9% (95% CI, 36.5-63.3%) for patients given >50 Gy (P = 0.67; HR, 1.33; 95% CI, 0.39-4.26). Higher doses did not achieved a higher survival from the current analysis.

Table 3 presents the incidence and sites of recurrence according to radiation doses and the irradiated field. Though no survival benefit was showed with higher doses, the cude regional recurrence rates tended to decrease with doses >50 Gy, but the difference was not statistically significant (P = 0.2). Two of 31 patients with doses >50 Gy and 3 of 20 patients with doses of ≤ 50 Gy developed pleura recurrences that higher dose may be benefitial for control of pleura recurrence. Meanwhile, two patients developed in-field recurrence. One of them was marginal recurrence with a dose of >50 Gy using 3D-CRT, the other one recurred in primary radiation field with a dose of 50 Gy using conventional RT. The crude local failure rates were low in both the two groups that a lower dose of 50 Gy may abtain a good local control.

### Treatment-related toxicity

Five patients (9.4%), comprising four who underwent conventional RT (7.5%) and one who received 3D-CRT/IMRT (1.9%), developed symptomatic radiation pneumonitis. One 69 year old patient with poor pulmonary function developed grade III acute severe pneumonitis (RTOG grading) which was exacerbated by bacterial lung infection when delivered dose of 28 Gy using conventional RT (he received no further dose of RT). This patient was treated using steroids and antibiotics and symptoms got relieved to a certain extent, but not complete remission. Finally, he died of radiation pneumonitis-related lung fibrosis 10 months later. The total dose to PTV for other 4 patients who developed grade II radiation pneumonitis was all 60 Gy. Three patients developed pericarditis after conventional RT, including two patients with grade I that required no treatment and one with grade II that required conservative treatment. One patient refused further treatment for grade IV radiation-related agranulocytosis when delivered dose of 28Gy.

## Discussion

The incidence of thymoma is rare, but it can present as a benign lesion or a highly infiltrative and even a metastasizing tumor [9]. Complete resection is crucial for stage III thymoma, despite the involvement of adjacent structures, and only 60% have a complete resection. Previous studies have reported the 5-yr and 10-yr OS rates of patients with stage III thymoma to be 60–80% and 50–70%, respectively [10-12]. Survival is improved after complete tumor resection compared with incomplete resection [1,2,13]. Adjuvant RT is generally used and is beneficial for patients with an incompletely resected tumor. However, definitive evidence regarding the benefit to patients of adjuvant radiotherapy after complete tumor resection are lacking, as there have been no prospective trials or higher quality retrospective studies.

Curran and colleagues recommended that adjuvant RT should be given for stage III thymoma after complete resection [2]. However, in their study, only four patients received postoperative RT, and therefore their results were less convincing. Eralp et al. also reported that adjuvant RT improved the OS and DFS rates for 36 patients with thymoma, but all stages of thymoma and both completely or incompletely resected tumors were included in their study [14]. However, Ruffini et al. reported the results of a series of 53 patients with stage III thymoma after complete resection, including 17 patients who were given postoperative RT and 36 who underwent surgery alone [15]. They observed higher recurrence rates and worse survival rates in patients who received adjuvant RT than surgery alone (P = 0.02). In a Japanese study, of 78 patients who received postoperative RT and 31 patients who underwent surgery alone after complete tumor resection, total recurrence rates were 23% and 26%, and local recurrence rates were 5.1% and 3.1%, respectively [12]. Adjuvant RT also conferred no benefit for patients with stage III tumor after complete resection. A meta-analysis reviewed data from 122 patients with stage III thymoma in order to determine the role of adjuvant RT after tumor resection in reducing recurrence and improving survival [16]. The risk of recurrence was 0–64% with adjuvant RT (53 patients) and 13–80% with S alone (69 patients) (P = 0.8, odd ratio = 0.87, 95% CI: 0.18–4.13). However, the heterogeneity among studies for stage III thymomas was significant (P = 0.02), and therefore the value of this meta-analysis study was not strong and the role of adjuvant RT was not confirmed. In the present study, the total recurrence rates had no significant decrease in patients with S + R compared with S alone, though adjuvant RT had a trend of reducing local recurrence (P = 0.09). These results were similar to those of previous studies that showed adjuvant RT had no significant effect on reducing the recurrence rate of stage III thymoma after complete resection [1,2,10-12,15,17].

In 2011, Patel and colleagues reported the results of 1,464 patients with a thymic malignancy from centers that participated in the Surveillance, Epidemiology, and End Results (SEER) program [18]. Among patients who did not receive RT, the 10-year OS rate was 41% compared with 42% for those who did receive RT (P = 0.06). The median OS for the patients who did not receive RT was 80 months compared to 97 months for those who did receive RT. In patients with Masaoka stage II–III malignancy, OS was significantly improved with RT (P = 0.002), and a trend in improved cause specific survival (CSS) was observed (P = 0.1). However, in patients who underwent total excision, the 5- and 10-year OS rates were 75% and 53%, respectively, for those who received RT, versus 71% and 55%, respectively, for those who did not (P = 0.1). The 5- year and 10-year CSS rates for those with a total excision were 93% and 83%, respectively, for patients who received RT, versus 92% and 78% for those who did not receive RT (P = 0.1). This report was lacking the definitive evidence of the benefit in patients after complete tumor resection for adjuvant radiotherapy, but there were benefit for all of the patients.

From the above retrospective studies, we can conclude that the role of adjuvant RT in patients with stage III thymoma after complete resection was not confirmed as a result of some limiting factors, including small sample sizes, a prolonged time for sample collection, different histological categories and different stages included in the same study, heterogeneity of data for multicenter studies, and the use of conventional RT alone. As a result, the subgroup analysis would be difficult due to variations in treatment and comparability between reports. Despite a large sample size the study by Patel et al., and the fact the SEER database is a national cancer registry for approximately 26% of the US population and 17 geographically defined registries between 1973 and 2003, similar problems also existed in this study [18]. In order to reduce the effect of these factors, we only selected patients with stage III thymoma who had undergone complete tumor resection in a single hospital for this analysis, but the bias due to the long duration required for data collection still resulted in the use of different radiation technologies (conventional RT vs. 3D-CRT/IMRT). Conventional RT often has a large radiation field, a poor accuracy for the target area and dose distribution, and high toxicity against normal tissues compared to 3D-CRT/IMRT. To date, there have been no reports regarding the use of 3D-CRT/IMRT as adjuvant radiotherapy in the treatment of thymomas.

In the present study, we performed further investigations on the effects of 3D-CRT/IMRT as an adjuvant therapy in patients with completely resected stage III thymoma. The results showed that patients with 3D-CRT/IMRT had a trend of improved 5-yr OS rate compared with conventional RT (P = 0.12), and an improved 5-yr OS rate when in pairwise compared with S alone (P = 0.049). However, when these three subgroups were compared in pooled, difference was not statistically significant (P = 0.2). Meantime, patients treated with 3D-CRT/IMRT compared with patients who received conventional RT had a trend of lower regional recurrence rate (3.6% vs. 32%). However, we should note there was long time span bias between the patients who were treated with 3D-CRT/IMRT compared with the conventional RT group. During this period, surgical skills have also improved in addition to radiotherapy techniques. Beyond that, the median time of follow-up in patients with 3D-CRT/IMRT was shorter than that in patients with conventional RT for the new radiation technique being introduced and used after the year 2000. Consequently, to further define this advantage, it will be necessary to conduct a strict and appropriately planned clinical trial to evaluate the utility of 3D-CRT/IMRT as an adjuvant treatment after the complete resection of thymoma.

In many reports, the doses of adjuvant RT for thymoma after complete resection were 30–60 Gy [19-22]. However, dose–response relationship in previous reports have not reached a consensus. Some researchers found that a higher dose did no good to tumor control and survival rates. Consequently, moderate doses of adjuvant RT was suggested [19,22]. Zhu et al. reported the outcomes of 128 thymomas treated with doses of 45–55 Gy. Five-year local control rates were similar between the doses of >50 Gy and ≤ 50 Gy [19]. A study of 103 patients with completely resected thymoma followed by radiotherapy reported by Ogawa et al. showed that a total dose of 40 Gy was effective in preventing mediastinal recurrence and no dose–response relationship was seen in intrathoracic control [22]. Conversely, Harnath et al. found in their study that doses in excess of 50 Gy were associated with significantly improved DFS and OS [21]. The similar result was found by Kundel et al. that postoperative RT to doses above 45 Gy may improve the DFS and OS of patients with invasive thymoma [23]. In our study, most of the failures occurred in the pleura, and the cude regional recurrence rates tended to decrease with doses >50 Gy, but the difference was not statistically significant (P = 0.2). No survival benefit was seen in patients of dose >50 Gy. Aboveall, controversy of dose–response relationship for adjuvant RT of thymoma still exists, and further evaluation by well-designed studies is required.

## Conclusions

Adjuvant 3D-CRT/IMRT showed potential advantages in improving survival and reducing relapse in patients with stage III thymoma after complete resection. However, for the cohort as a whole, adjuvant RT did not significantly improve survival or reduce recurrence. Doses of ≤ 50 Gy may be effective and could be prescribed for adjuvant RT. To confirm the role of adjuvant 3D-CRT/IMRT in patients who undergo a complete resection of thymoma, a multicenter randomized study should be performed.

## Competing interests

The authors declare that they have no competing interests.

## Author’s contributions

CF drafted the manuscript. CF, QF,YC and YZ participated in data collection, and helped to analyze the data. QF conceived of the study, participated in its design. All authors made substantial contributions to acquisition and statistical analysis of data, and read and approved the final manuscript.
